# Supplementary material for: Graph lesion-deficit mapping of fluid intelligence
Source: Brain. 2022 Dec 28;146(1):167–81. doi: 10.1093/brain/awac304 (PMC9825598; doi:10.1093/brain/awac304)
Supplement: awac304_Supplementary_Data [file awac304_supplementary_data.pdf]

Graph lesion-deficit mapping of fluid intelligence

Lisa Cipolotti,[1,2*] James K Ruffle,[2,3] Joe Mole,[1,2] Tianbo Xu,[2] Harpreet Hyare,[2,3] Tim Shallice,[4,5] Edgar Chan[1,2] and Parashkev Nachev[2]

Supplementary Material

Stochastic block models

A stochastic block model (SBM)[1] is a generative model of the community structure of a graph composed of N nodes, divided into B blocks with edges e_{rs} between blocks r and s. The model can be framed hierarchically, where edge counts e_{rs} form block multigraphs with nodes corresponding to individual blocks and edge counts arising as edge multiplicities between block pairs, including self-loops. We seek to infer the most plausible partition $\{b_i\}$ of the nodes, where $\{b_i\} \in [1, B]^N$ identifies the block membership of node i in observed network G, with maximisation of the posterior likelihood $P(G|\{b_i\})$. The result is a hierarchically organised community structure of nodes assigned into blocks that yields the most compact representation of the graph, as indexed by its minimum description length[2], $\sum$. The general approach is described in further detail elsewhere[1].

An extension to the hierarchical SBM is its layered formulation, permitting modelling of a graph structure where edges reflect disparate forms of interaction[3]. In the context of lesion deficit mapping, we can use a layered SBM to model relations between voxels driven by two distinct effects: the underlying neural dependence of the function of interest and the pathological structure of the lesions used to map it. Key here is formal comparison between models that encode these effects separately, within their own layers, vs those where the distinction is not respected. In a Bayesian setting[3], the procedure for model selection amounts to finding the model maximising posterior likelihood as

$$P(\{\theta\}|\{G_l\}) = \frac{P(\{G_l\}|\{\theta\})P(\{\theta\})}{P(\{G_l\})},$$

where $\{\theta\}$ denotes the shorthand for the model parameters. In our case, $\{\theta\} = \{\{b_i\}, \{e_{rs}^l\}\}$, where N nodes are divided into B blocks via the membership vector $\{b_i\} \in [1, B]^N$, and the distribution of covariates in edges in groups r and s is given by the edge counts e_{rs}, with e_{rs}^l corresponding to the former at a given layer. $P(\{\theta\})$ is the prior probability on these parameters, with $P(\{G_l\})$ corresponding to the normalisation constant. The approach is further detailed by Peixoto[3], formulating the most succinct representation of the data as one with the minimum description length[2], $\sum$. Since the prior probabilities are nonparametric, the procedure also becomes parameter-free.

Choosing the model with the smallest description length $\sum$ is the means of balancing model complexity and goodness of fit[2]. We consider two candidate models throughout our experimental design: model $\mathcal{H}_a$, where layers are true descriptors corresponding to the weighted edges of our deficit of interest in one layer and the connection matrix of the set of lesions in another layer, and a null model $\mathcal{H}_b$ where the edges describing deficit and the lesion connectivity effects are randomly interspersed across layers. The comparative magnitude of the description length of each model yields the following posterior odds ratio:

$$\Lambda = \frac{P(\{\theta\}_a|\{G_l\},\mathcal{H}_a)P(\mathcal{H}_a)}{P(\{\theta\}_b|\{G_l\},\mathcal{H}_b)P(\mathcal{H}_b)},$$

simplifying to

$$\Lambda = \exp(-\Delta\textstyle\sum)\frac{P(\mathcal{H}_a)}{P(\mathcal{H}_b)}.$$

In this instance, $P(\{\theta\}|\{G_l\}, \mathcal{H})$ is the posterior according to a given hypothesis $\mathcal{H}$, i.e., the true or null layered formulation. $P(\mathcal{H})$ is then the prior belief for hypothesis $\mathcal{H}$, and $\Delta\sum = \sum_a - \sum_b$ the difference in the model description length for these hypotheses.

Semi-synthetic evaluation of stochastic block model lesion-deficit mapping

The application of stochastic block modelling (SBM) to lesion-deficit inference is underwritten by the validity of the underlying statistical framework. No purely empirical external validation is possible here because the functional anatomy of the neural substrate is definitionally unknown—it is what we are using lesion-deficit models to infer—and discriminative models of outcome need only identify the anatomical boundaries—jointly defined by lesion and neural effects—that determine outcomes. But we can establish a semi-synthetic validation framework, where an array of plausible, empirically-guided anatomical ground truths are hypothetically posited, and the fidelity of candidate models of real lesions used to retrieve them is explicitly quantified[4]. Here we derive ground truths from the large-scale meta-analytic repository NeuroQuery[5], extracting functional maps of terms selected to span a broad range of cognitive domains, and exhibit widely distributed neuroanatomical patterns. The terms used were “action”, “aversion”, “language”, “mood”, “motor”, and “sensation”. Each map was re-sampled into the same $4mm^3$ isotropic space employed in the SBM pipeline, and thresholded at a conservative Z score of >=4. The connected components of each mask where identified, and clusters smaller than 27 voxels or sampled by fewer than 3 lesions were removed. Six sets of hypothetical continuous lesion-deficit relations were then created by summing over the intersection between each lesion segmentation and each corresponding ground truth, yielding a weighted “deficit score” for each patient and each functional domain.

We proceeded to evaluate these semi-synthetic lesion-deficit relations with a non-parametric Bayesian hierarchical weighted stochastic block model incorporating layered and attributed properties, implemented in graph-tool (https://graph-tool.skewed.de)[2,3,6-8], exactly as in the main analysis. We began by fitting a null model, with the two kinds of edge weight—the deficit score and the lesion co-occurrence—randomly distributed across two layers. We then fitted a test model where each type of weight was consistently assigned to its own layer. Deficit weights were modelled as Gaussian; lesion co-occurrence weights as Poisson distributions. Having initialised a fit, we used simulated annealing to further optimise it, with 1000 iterations and a default inverse temperature of 1 to 10. We used model entropy to determine if the layered model fit was better than the null, indicating that the inferred community structure corresponded to the synthetic ground truth and lesion co-occurrence effects. To visualise the inferred communities, we backprojected the incident edge weights onto the brain, deriving the mean and 95% credible intervals. Bayesian model comparison based on minimum description length was used to determine if the layered models were more plausible than the null (Supplementary Figure 4). To show the relationship between inferred deficit and lesion co-occurrence edge weights, we extracted the posterior means whose 95% credible interval did not cross zero in each test model and plotted their correlation.

To compare the fidelity of our anatomical retrieval to that achievable with conventional lesion-deficit mapping, we performed standard mass-univariate voxel-wise lesion-deficit mapping (VLSM) of the same data implemented in SPM. The lesions were smoothed with a Gaussian kernel of 4mm full-width at half-maximum to facilitate voxel-wise spatial inference within a random Gaussian fields framework, and entered into a voxel-wise general linear model with the lesion intensity as the dependent variable and the deficit score as the independent variable. The resultant statistical maps were thresholded at $p<0.05$ family-wise error corrected. Models omitting the smoothing step yielded very similar maps (data not shown). The comparative fidelity of SBM vs VLSM models of the same data was then quantified by the difference in the Dice score for each inferred map relative to the ground truth. Note that the complex spatial structure of the ground truth maps employed here, chosen to provide the most robust test of the retrieval of distributed neural substrates, precludes interpretation of the absolute Dice score: the focus here is on the comparison with conventional topological models.

The SBM models were quantitatively superior to VLSM across the entire set ($p = 0.028$, Supplementary Figure 5). The qualitative results, visualised in the same figure, demonstrate the vulnerability of VLSM models to spatial biases driven by lesion morphology, and the ability of SBM models to resist them.

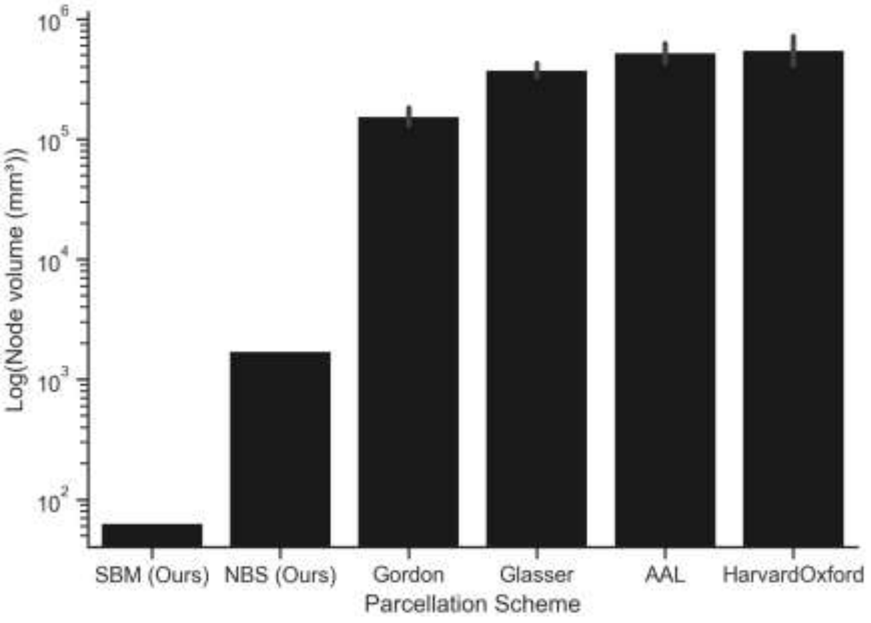

Supplementary Figure 1. Bar plot of the mean spatial unit of analysis volumes of our stochastic block (SBM) and network based statistics (NBS) models compared with alternative regional parcellation schemes. Note our volumes are substantially smaller. The errors identify 95% confidence intervals. Note the ordinate is decimal log transformed.

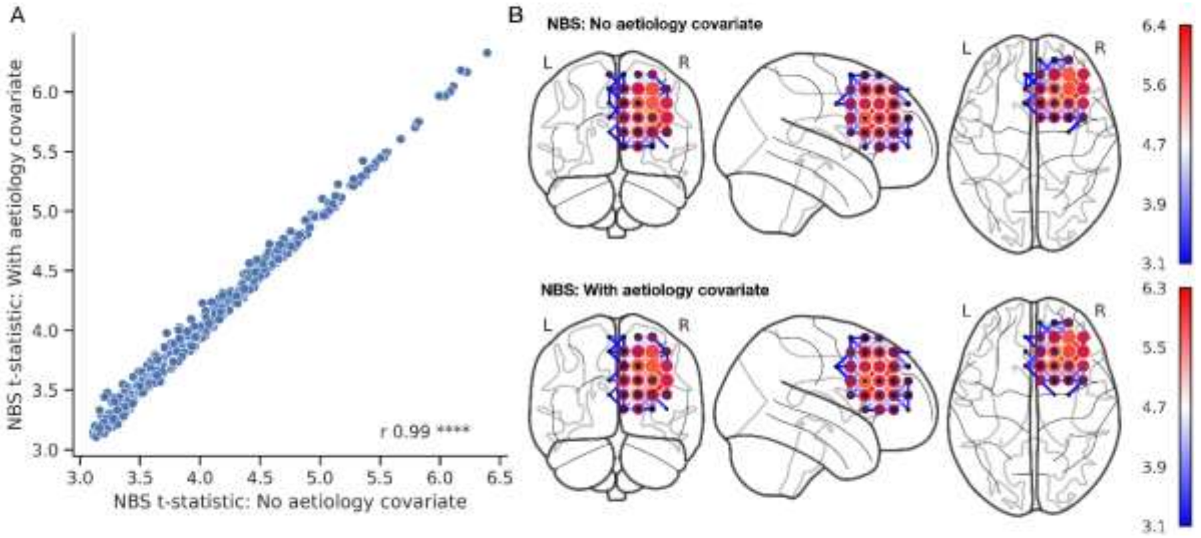

Supplementary Figure 2. A. Scatter plot of significant edges derived from network-based statistics models including (ordinate) and excluding (abscissa) a lesion aetiology covariate. Note all points lie very close to the diagonal, indicating a minimal difference between the two. B. Visual connectome plots of the network-based statistics models excluding (top) and including (bottom) the aetiology covariate.

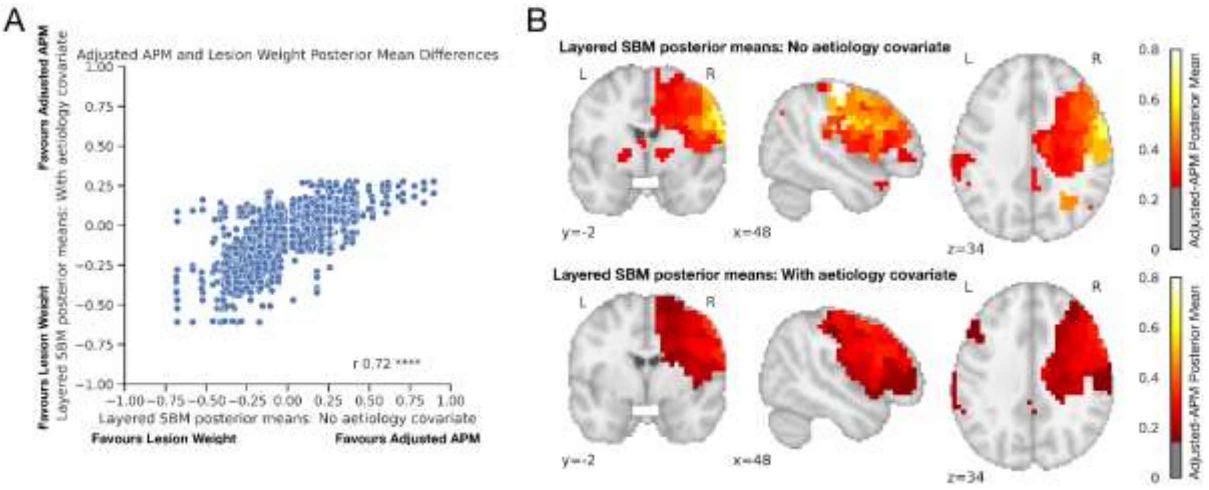

Supplementary Figure 3. A. Scatter plot of the relation between posterior means of community blocks of stochastic block models with (ordinate) and without (abscissa) an aetiology layer. Note the former exhibits a narrower range of APM-related variation, suggesting weaker disentanglement from co-occurrence. B. Visualisation of the posterior means of the SBM without (top) and with (bottom) inclusion of aetiology in a separate layer. The description length of the more complex model was ~2.4 times that of the simpler model (881118.22 vs 2133947.48 nats), a dramatic rise in description length for only a single additional feature, suggesting the former is overparameterised.

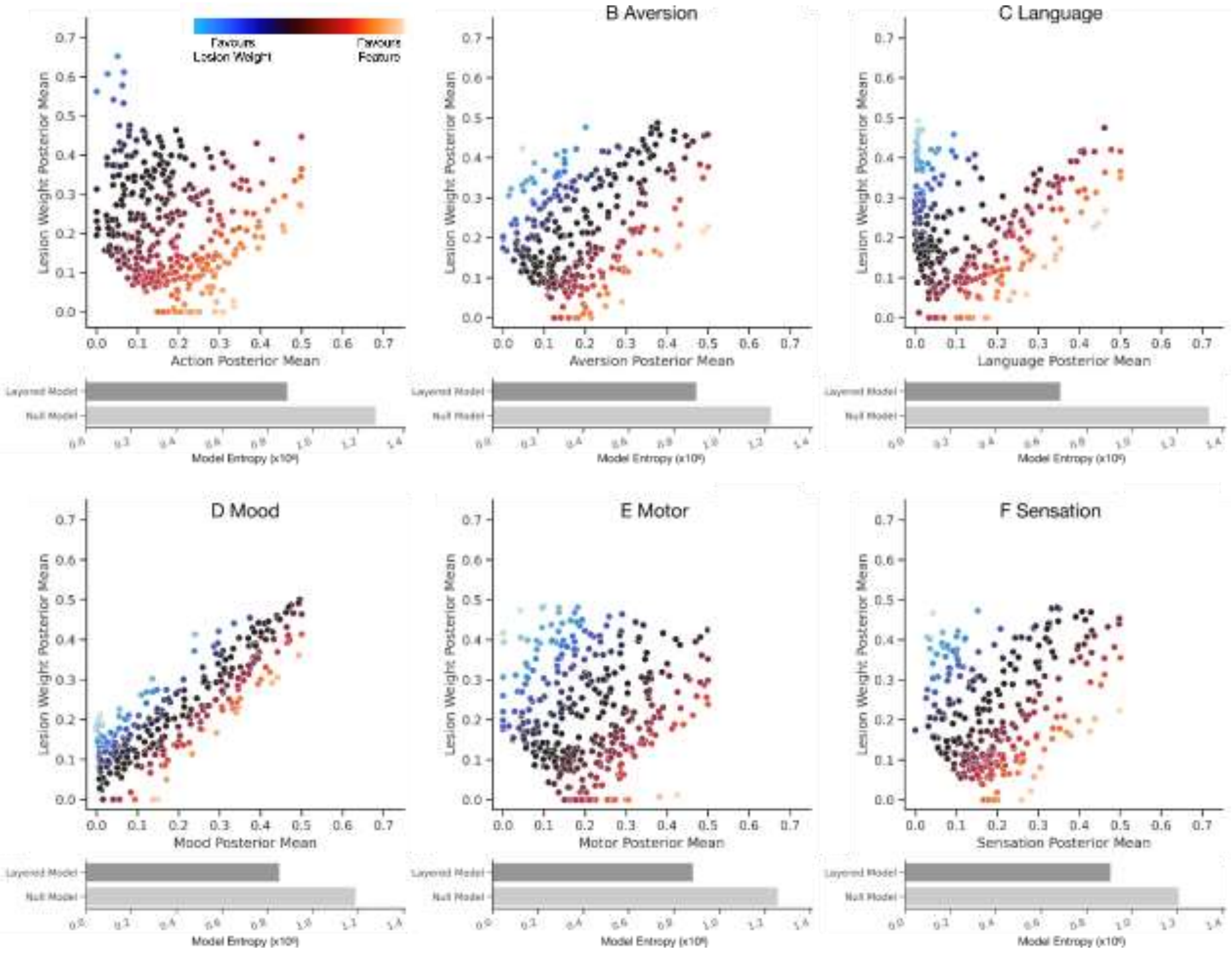

Supplementary Figure 4. Scatter plots of the correlation between lesion co-occurrence (ordinate, red-yellow) and deficit (abscissa, blue-light blue) posterior means from the blocks of the test SBM models in each of the six ground truth experiments. Each individual point corresponds to a block at the l_0 hierarchical layer. Below each plot is a bar chart of the corresponding test (dark grey) and null (light grey) model entropies in nats. Note the disentanglement of lesion co-occurrence and deficit effects, and lower entropies for the layered models across the set, indicating superiority. The entropy difference, x, between the layered and null formulation translates to a posterior odds ratio of e^x for the layered formulation over the non-layered alternative, as is further detailed in the supplementary methods.

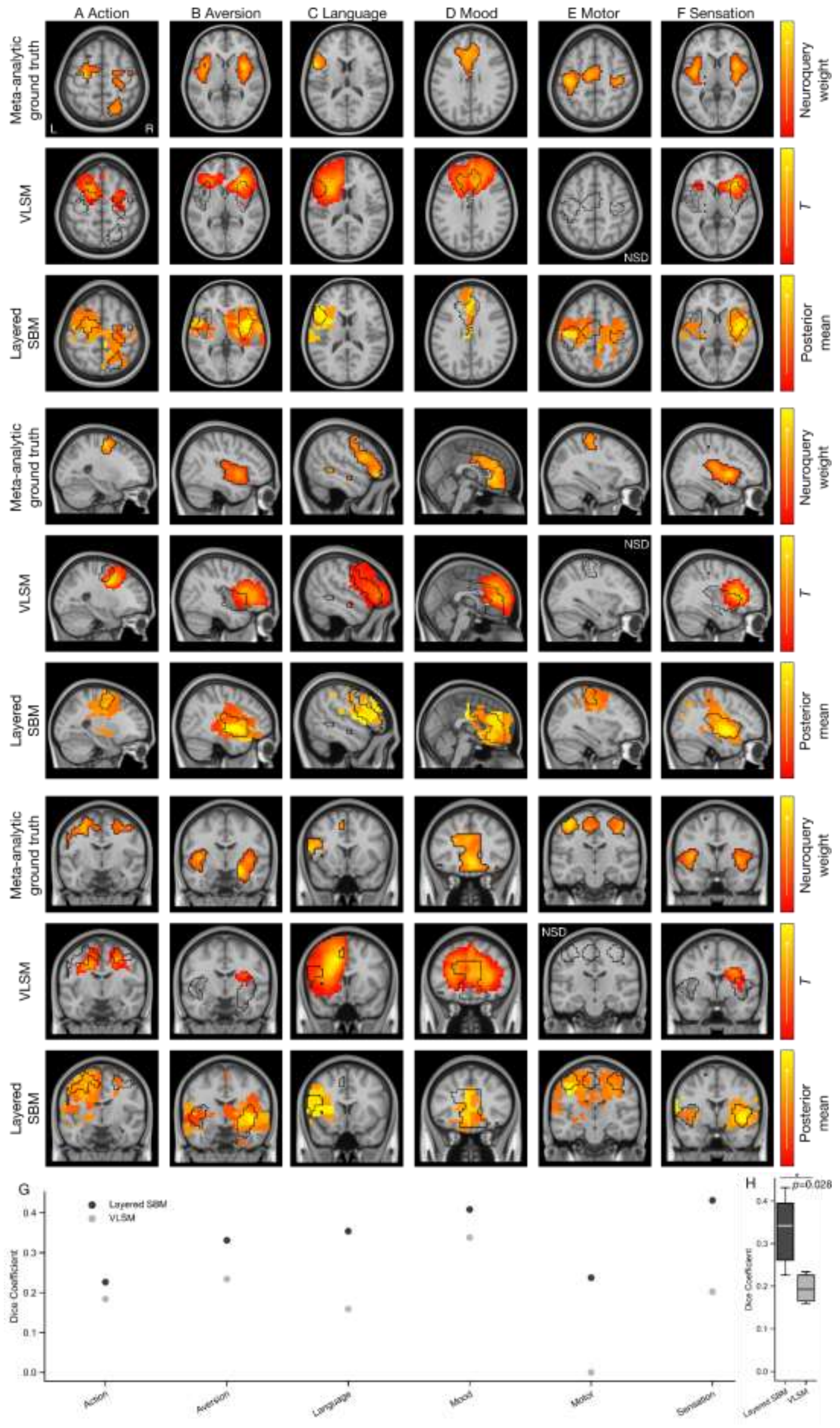

Supplementary Figure 5. A-F. Triplanar view of the comparison between VLSM (rows 2, 5, and 8) and SBM (rows 3, 6, and 9) inference across the six domains, with the ground truth outlined in black over the source NeuroQuery weights (rows 1, 4 and 7). Note that VLSM retained no significant results for identifying the motor ground truth. G. Dice scores for SBM (black) and VLSM (grey) ground truth retrieval. H. Dice score boxplots across the set, with the p value for the difference.

References

1. Peixoto TP. Hierarchical Block Structures and High-Resolution Model Selection in Large Networks. *Physical Review X*. 03/24/ 2014;4(1):011047. doi:10.1103/PhysRevX.4.011047
2. Peixoto TP. Entropy of stochastic blockmodel ensembles. *Physical Review E*. 05/30/ 2012;85(5):056122. doi:10.1103/PhysRevE.85.056122
3. Peixoto TP. Inferring the mesoscale structure of layered, edge-valued, and time-varying networks. *Physical Review E*. 10/09/ 2015;92(4):042807. doi:10.1103/PhysRevE.92.042807
4. Mah YH, Husain M, Rees G, Nachev P. Human brain lesion-deficit inference remapped. *Brain*. Sep 2014;137(Pt 9):2522-31. doi:10.1093/brain/awu164
5. Dockes J, Poldrack RA, Primet R*, et al*. NeuroQuery, comprehensive meta-analysis of human brain mapping. *Elife*. Mar 4 2020;9doi:10.7554/eLife.53385
6. Peixoto TP. Nonparametric weighted stochastic block models. *Physical Review E*. 01/16/ 2018;97(1):012306. doi:10.1103/PhysRevE.97.012306
7. Peixoto TP. Merge-split Markov chain Monte Carlo for community detection. *Physical Review E*. 07/13/ 2020;102(1):012305. doi:10.1103/PhysRevE.102.012305
8. Peixoto TP. The graph-tool python library. *figshare*. 2014;doi:10.6084/m9.figshare.1164194
